# Sialyltransferase ST6GAL-1 mediates resistance to chemoradiation in rectal cancer

**DOI:** 10.1016/j.jbc.2022.101594

**Published:** 2022-01-15

**Authors:** Mary Smithson, Regina Irwin, Gregory Williams, Katie L. Alexander, Lesley E. Smythies, Marie Nearing, M. Chandler McLeod, Sameer Al Diffalha, Susan L. Bellis, Karin M. Hardiman

**Affiliations:** 1 Department of Surgery, University of Alabama at Birmingham, Birmingham, Alabama, USA; 2 Department of Medicine, University of Alabama at Birmingham, Birmingham, Alabama, USA; 3 Department of Pathology, University of Alabama at Birmingham, Birmingham, Alabama, USA; 4 Department of Cell, Developmental, and Integrative Biology, University of Alabama at Birmingham, Birmingham, Alabama, USA; 5 Department of Surgery, Birmingham Veterans Affairs Medical Center, Birmingham, Alabama, USA

**Keywords:** ST6GAL-1, radiation biology, sialyltransferase, cancer biology, resistance, 5-FU, 5-fluorouracil, ATCC, American Type Culture Collection, CI, confidence interval, CV, control vector, DMEM, Dulbecco's modified Eagle's medium, FACS, fluorescence-activated cell sorting, FBS, fetal bovine serum, IgG, immunoglobulin G, KD, knockdown, PDX, patient-derived xenograft, qPCR, quantitative PCR, Sia, sialic acid, SNA, *Sambucus nigra*, SNL, *Sambucus nigra* lectin, TNFR1, tumor necrosis factor receptor 1

## Abstract

Locally advanced rectal cancer is typically treated with chemoradiotherapy followed by surgery. Most patients do not display a complete response to chemoradiotherapy, but resistance mechanisms are poorly understood. ST6GAL-1 is a sialyltransferase that adds the negatively charged sugar, sialic acid (Sia), to cell surface proteins in the Golgi, altering their function. We therefore hypothesized that ST6GAL-1 could mediate resistance to chemoradiation in rectal cancer by inhibiting apoptosis. Patient-derived xenograft and organoid models of rectal cancer and rectal cancer cell lines were assessed for ST6GAL-1 protein with and without chemoradiation treatment. *ST6GAL-1* mRNA was assessed in untreated human rectal adenocarcinoma by PCR assays. Samples were further assessed by Western blotting, Caspase-Glo apoptosis assays, and colony formation assays. The presence of functional ST6GAL-1 was assessed *via* flow cytometry using the *Sambucus nigra* lectin, which specifically binds cell surface α2,6-linked Sia, and *via* lectin precipitation. In patient-derived xenograft models of rectal cancer, we found that ST6GAL-1 protein was increased after chemoradiation in a subset of samples. Rectal cancer cell lines demonstrated increased ST6GAL-1 protein and cell surface Sia after chemoradiation. ST6GAL-1 was also increased in rectal cancer organoids after treatment. ST6GAL-1 knockdown in rectal cancer cell lines resulted in increased apoptosis and decreased survival after treatment. We concluded that ST6GAL-1 promotes resistance to chemoradiotherapy by inhibiting apoptosis in rectal cancer cell lines. More research will be needed to further elucidate the importance and mechanism of ST6GAL-1-mediated resistance.

Rectal cancer and other gastrointestinal cancers, including gastric and esophageal cancers, are treated with chemoradiation including 5-fluorouracil (5-FU), or its oral equivalent capecitabine, and radiation daily for 6 weeks followed by surgery. Rectal cancer accounts for one-third of all colorectal cancer cases with approximately 45,000 Americans diagnosed annually, and this rate is increasing (1). Around 15% of patients are complete responders to preoperative chemoradiotherapy, and clinical trials managing these complete responders without surgery are ongoing (2). While this new management is promising, most patients are not complete responders, and thus investigations into the mechanisms of resistance to treatment are vital (3, 4). ST6GAL-1 is the primary enzyme responsible for α2-6 sialylation of N-glycans on select glycoproteins. Altered glycosylation is a hallmark of cancer (5, 6), and increased ST6GAL-1 mRNA and protein have been found in multiple types of cancer, including ovarian, pancreatic, and colonic (7, 8, 9, 10, 11) cancers. High ST6GAL-1 has been associated with increased metastatic potential and poor survival (9, 11, 12). ST6GAL-1 adds the negatively charged sugar, sialic acid (Sia), to certain proteins in the Golgi, which alters protein function (8, 13, 14). Multiple affected proteins have been described including tumor necrosis factor receptor 1 (TNFR1), Fas receptor, epidermal growth factor receptor, and β1-integrin (7, 8, 13, 15, 16). TNFR1 and Fas are cell surface receptors that mediate apoptosis, and prior studies have found that α2-6 sialylation decreases propagation of apoptotic signaling. We hypothesized that ST6GAL-1 may mediate resistance to chemoradiation in rectal adenocarcinoma by decreasing apoptosis.

There is a critical need for improved therapies in rectal cancer in order to enhance survival and potentially decrease morbidity by improving the rate of complete response to chemoradiotherapy. In this study, we investigated a novel role for ST6GAL-1 in therapeutic resistance in rectal cancer. We generated and treated three patient-derived xenograft models of rectal cancer with chemoradiation and found that ST6GAL-1 protein was increased in two out of three of the models. We then utilized human rectal cancer samples to assess the prevalence and distribution of ST6GAL-1 in untreated tumors. We also found that ST6GAL-1 was increased after treatment in primary rectal cancer organoids. We subsequently used rectal cancer cell lines to assess functional ST6GAL-1 protein after chemoradiotherapy and found that it was increased. In addition, we transduced rectal cancer cell lines with shRNA against ST6GAL-1 (knockdown [KD]) in order to study the effects of ST6GAL-1 on post-treatment cell survival and apoptosis. We showed that ST6GAL-1 mediates therapeutic resistance in rectal cancer by decreasing apoptosis and TNFR1 as the potential target of this decrease. Taken together, these studies implicate ST6GAL-1 as a potentially important mediator of resistance to chemoradiation in rectal cancer.

## Results

### ST6GAL-1 in chemoradiation-treated patient-derived xenograft models

**Figure 1:**
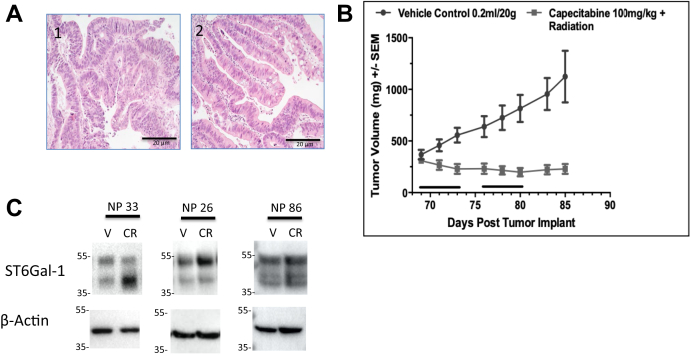
**ST6Gal-1 is increased in some rectal cancer patient-derived xenograft (PDX) models after chemoradiation***A*, H&E staining of PDX (2), compared with the original tumor material obtained at the time of biopsy (1), shows similar histology. *B*, decreased tumor volume compared with vehicle in mice treated with capecitabine and radiation (*black bars* indicate treatment days) when PDX models are implanted in the flank and treated for 2 weeks. *C*, ST6Gal-1 increases after chemoradiation in two of three treated PDX models compared with vehicle (NP33: 2.40-fold increase, N = 4, *p* = 0.01; NP26: 2.18-fold increase, N = 3, *p* = 0.01; NP86: 0.88-fold decrease, N = 3, *p* = 0.33).

Three patient-derived xenograft (PDX) models were assessed: NP26, NP86, and NP33, which were created from pretreatment biopsy samples from consented rectal cancer patients. Once tumors reached appropriate size in Central Institute for Experimental Animals NOD/Shi-scid IL2gamma(null) (NOG) mice, they were implanted into the flank of nude mice. Histology images show that PDX tumors were similar to their tumor of origin (Fig. 1*A*). Mice implanted with tumors were treated with capecitabine 100 mg/kg (oral equivalent of 5-FU) and radiation or vehicle. Tumors undergoing chemoradiation were significantly smaller in volume compared with vehicle treatment (Fig. 1*B*). After 2 weeks of treatment and waiting period of 1 week, tumors were harvested and protein was collected. ST6GAL-1 protein was assessed by Western blotting and found to be increased in two of the three models (Fig. 1*C*). Upper band in Western blot is whole ST6GAL-1, and lower band is cleaved ST6GAL-1; quantification included both bands. In two of the three models, ST6GAL-1 increased significantly after chemoradiation. In NP33, there was an increase from 1.01-fold to 2.40-fold from vehicle to treated (N = 4, *p* = 0.01); NP26 showed an increase from 1.07-fold to 2.19-fold from vehicle to treated (N = 3, *p* = 0.01); and NP86 showed a nonsignificant decrease from 1.32-fold to 0.88-fold from vehicle to treated (N = 3, *p* = 0.33).

### ST6GAL-1 expression in human rectal cancers

**Figure 2:**
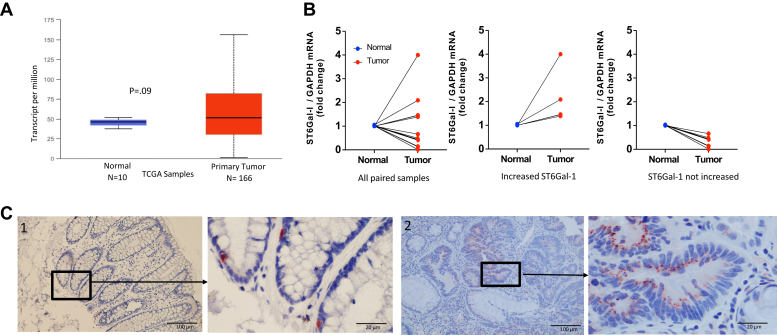
**ST6Gal-1 expression is heterogenous in rectal cancer.***A*, in The Cancer Genome Atlas (TCGA) samples, ST6Gal-1 transcripts from normal rectum compared with tumor samples (unpaired) identified heterogeneity across untreated rectal cancer sample. *B*, PCR on rectal cancer tumor-normal pairs revealed variability in ST6Gal-1 mRNA in untreated samples where some have increased ST6Gal-1 in the tumor compared with normal and others do not. *C*, immunostaining for ST6Gal-1 protein whereby the location of ST6Gal-1 differs between normal rectum and cancer. 1: Normal human rectum with ST6Gal-1 confined only to some of the crypt cells. 2: Untreated human rectal cancer with ST6Gal-1 in a subpopulation of the adenocarcinoma cells.

In order to ascertain how commonly ST6GAL-1 mRNA is increased in untreated human rectal samples, the UALCAN database (ualcan.path.uab.edu/index.html) (17) was used to query transcripts in human rectal adenocarcinomas from The Cancer Genome Atlas and the level of ST6GAL-1 transcripts comparing 166 untreated primary rectal adenocarcinoma samples to ten unmatched samples of normal rectum and found that the increase was not statistically different. The large standard deviation for the tumors indicates heterogeneity in expression of ST6GAL-1 mRNA between tumors (Fig. 2*A*). Because these data contained so few normal samples and the comparisons were not matched, we performed quantitative PCR (qPCR) on 12 pretreatment rectal cancer biopsy specimens and their matched normal rectal tissue. We again found heterogeneity between tumors whereby four tumors showed an increase in ST6GAL-1 mRNA compared with matched normal rectum, whereas the other eight samples did not have increased ST6GAL-1 mRNA compared with matched normal samples (Fig. 2*B*). In order to assess the distribution of ST6GAL-1 protein within individual tumors, we performed immunostaining in untreated human rectal adenocarcinoma samples and normal rectal tissues. We found that ST6GAL-1 protein was only in a subset of cells within the base of the crypts (stem cell compartment) of the normal tissues (similar to prior work; Fig. 2*C*), but that in the human rectal adenocarcinoma samples, ST6GAL-1 was in some but not in all the adenocarcinoma cells in any given tumor (Fig. 2*C*). The number of cells staining positive for ST6GAL-1 between tumors appeared variable but was not quantified.

### Functional ST6GAL-1 increases after radiation in rectal cancer cell lines and organoids

**Figure 3:**
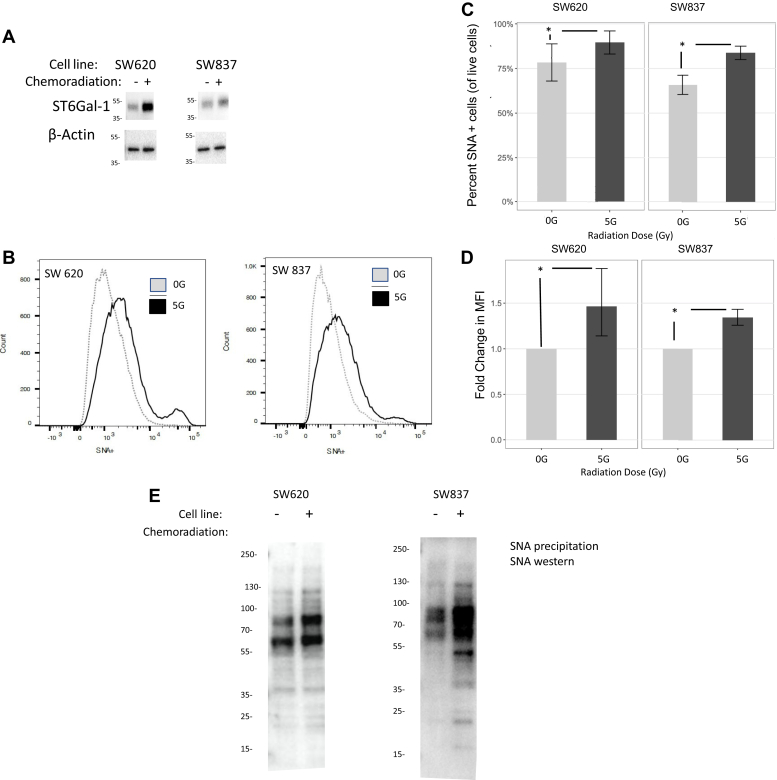
**Functional ST6Gal-1 protein is increased after chemoradiation in rectal cancer cell lines.***A*, ST6Gal-1 protein is increased in cell lines after chemoradiation (SW620: 1.69-fold increase, N = 3, *p* = 0.0001 and SW837: 1.60-fold increase, N = 3, *p* = 0.004). *B*, Sia is increased in chemoradiation-treated rectal cancer cell lines compared with vehicle by flow cytometry. *C*, quantification of increased percent of SNA-positive cells after chemoradiation (SW620 and SW837, N = 4, ∗ indicates *p* < 0.05). *D*, quantification of mean fluorescent intensity in SNA after chemoradiation (SW620 and SW837, N = 4, ∗ indicates *p* < 0.05). *E*, precipitation with SNA beads followed by Western blot for SNA was performed in untreated SW620 and SW837 cells and 24 h after chemoradiation and identified increased sialylation of proteins after treatment (N = 3). Sia, sialic acid; SNA, *Sambucus nigra*.

SW620 and SW837 rectal cancer cell lines were plated and treated 24 h later with 5-FU and radiation (5 Gy). Protein was collected 5 days after treatment. ST6GAL-1 protein increased after chemoradiation (SW620: 1.69-fold increase, *p* ≤ 0.0001, N = 3 and SW837: 1.60-fold increase, *p* = 0.004, N = 3) (Fig. 3*A*). In order to assess whether the increased ST6GAL-1 was functional, treated and untreated cells were assessed for ST6GAL-1 activity using an FITC-labeled *Sambucus nigra* (SNA) lectin (SNL), which binds to the Sia on cell surface proteins that have been added by ST6GAL-1 in the Golgi. These cells were assessed *via* flow cytometry for SNA positivity. The number of cells staining positive for SNA increased in both cell lines after treatment (SW620: odds ratio = 1.93 [95% confidence interval (95% CI): 1.89–1.97], *p* < 0.001 and SW837: odds ratio = 2.69 [95% CI: 2.65–2.74], *p* < 0.001, N = 4 separate experiments per cell line) (Fig. 3, *B* and *C*). In addition, mean fluorescent intensity also increased in both cell lines after chemoradiation treatment (SW620: fold change of 1.46, *p* ≤ 0.01 and SW837: fold change of 1.34, *p* < 0.002, N = 4) (Fig. 3*D*) indicating that the function of ST6GAL-1 also increases after treatment. SW837 and SW620 cell lines were treated with chemoradiation (3 μM of 5-FU and 5 Gy radiation) and then underwent precipitation with SNA-agarose, followed by SNA lectin blotting where equal amounts of total protein were added to the gel (N = 3), in order to identify all sialylated proteins. Sialylation (addition of Sia to cell surface proteins by ST6GAL-1) increased after chemoradiation compared with vehicle (Fig. 3*E*).

**Figure 4:**
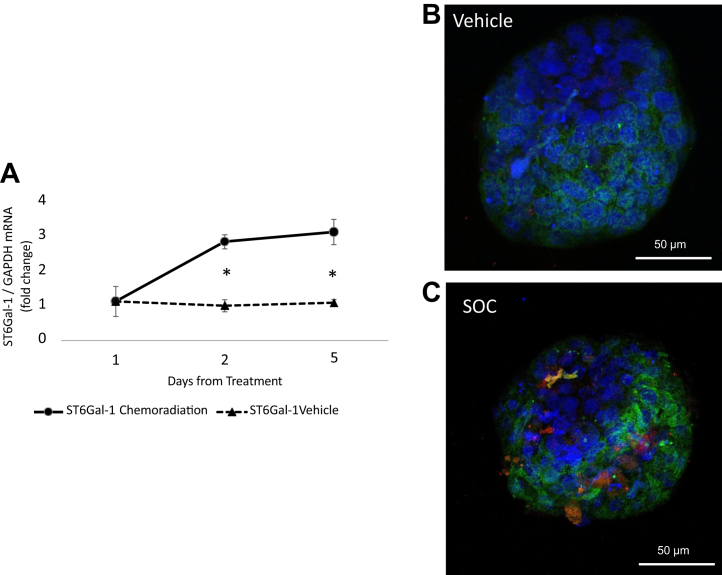
**ST6Gal-1 and Sia increase in primary rectal cancer organoids after treatment.***A*, ST6Gal-1 mRNA increases 3.1-fold at 5 days after chemoradiation treatment of primary rectal cancer organoids compared with 1.1-fold increase in vehicle (N = 3, *p* = 0.007). *B*, immunofluorescent staining of vehicle organoid (ST6Gal-1: *red* and Sia: *green*). *C*, increased ST6Gal-1 (*red*) and Sia (*green*) is seen 5 days after chemoradiation. Sia, sialic acid.

We also assessed whether ST6GAL-1 increases after treatment in rectal cancer organoids grown from primary rectal cancer tissue by both PCR and immunofluorescent staining. PCR for ST6GAL-1 revealed an increase after treatment. For example, there was a 3.1-fold increase in ST6GAL-1 on day 5 after chemoradiation (3 μM 5-FU, 5 Gy radiation) compared with a 1.1-fold increase in vehicle (N = 3, *p* = 0.007; Fig. 4*A*). We also assessed changes in fluorescent staining of ST6GAL-1 (*red*) and Sia (*green*) after chemoradiation (3 μM 5-FU, 5 Gy radiation) in rectal cancer organoids and found that both were increased in the treated samples (Fig. 4, *B* and *C*).

### ST6GAL-1 mediates treatment resistance

**Figure 5:**
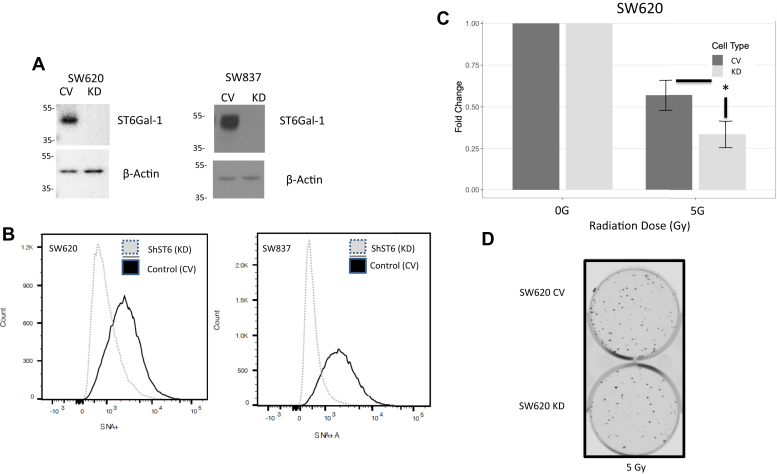
**Knockdown (KD) of ST6Gal-1 decreases cell viability after chemoradiation treatment.***A*, Western blot confirming ST6Gal-1 KD in SW620 and SW837 cell lines transduced with control vector (CV) or shST6Gal-1 (KD) lentivirus. *B*, decreased SNA in KD cells compared with CV cells by flow cytometry confirming loss of functional ST6Gal-1. *C*, increased fold change in colony formation in CV cells compared with ST6Gal-1 KD cells after chemoradiation indicating ST6Gal-1 mediates therapeutic resistance (SW620, N = 3, ∗ indicates *p* < 0.05). *D*, example plates for colony formation assay comparing post-treatment CV *versus* KD showing more colonies on CV compared with KD plates.

SW620 and SW837 cells were transduced with either a control vector (CV) lentivirus (nonmammalian shRNA, CV) or lentivirus encoding shRNA for ST6GAL-1 (KD). Cells were incubated, colonies were puromycin selected, and Western blots confirmed successful KD of ST6GAL-1 protein in both cell lines (Fig. 5*A*). SW620 CV and KD and SW837 CV and KD were stained with FITC-labeled SNA and evaluated for surface Sia by flow cytometry indicative of functional ST6GAL-1. As shown in Figure 5*B*, the CV cells have more SNA+ cells than the KD cells in each cell line, again indicating successful KD of functional ST6GAL-1. To assess whether the previously seen increase in ST6GAL-1 was causing treatment resistance, SW620 CV and KD cells were treated with chemoradiation to assess for differences in survival *via* colony formation assay at 5 days after treatment. As shown in Figure 5, *C* and *D*, cell survival was decreased in the treated ST6GAL-1 KD cells by approximately 50%, further supporting that ST6GAL-1 is mediating resistance, indicating that resistance is mediated by ST6GAL-1 (SW620: *beta* [log fold change 5 Gy over 0 Gy] = −0.54 [95% CI: −0.84, −0.25], N = 3 experiments each with three technical replicates, *p* ≤ 0.001).

### ST6GAL-1-dependent decrease in apoptosis

**Figure 6:**
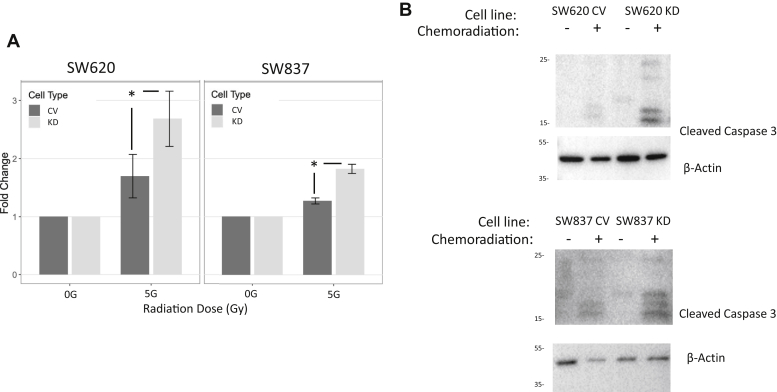
**ST6Gal-1 causes decreased apoptosis in rectal cancer cells after chemoradiation.***A*, caspase 3/7 Glo assay 24 h after treatment with chemoradiation revealed increased apoptosis, represented by fold change of relative light units, after treatment in the ST6Gal-1 KD cells compared with CV cells (SW620 and SW837, N = 5, ∗ indicates *p* < 0.05). *B*, Western blot for cleaved caspase 3 in SW620 and SW837 cells 24 h after treatment revealed increased cleaved caspase 3, indicating apoptosis, in the KD cells after chemoradiation compared with CV cells (N = 3).

Apoptosis is the main mechanism of cell death following chemoradiation treatment of adenocarcinomas. We hypothesized that the resistance to chemoradiation we identified in the rectal cancer cell lines was mediated by decreased apoptosis likely *via* sialylation of TNFR or Fas death receptors. SW620 CV and KD and SW837 CV and KD cells were plated and treated with chemoradiation, and 24 h later, Caspase 3/7 assay was performed to measure apoptosis. Comparing ST6GAL-1 KD cells to CV cells, we identified a 1.58-fold and 1.45-fold increase in apoptosis for SW620 and SW837, respectively (SW620: *beta* [log fold change 5 Gy over 0 Gy] = 0.46 [95% CI: 0.34–0.58], *p* < 0.001 and SW837: *beta* [log fold change 5 Gy over 0 Gy] = 0.37 [95% CI: 0.27–0.47], *p* < 0.001; N = 3 experiments) indicating that ST6GAL-1 is protecting cells from apoptosis (Fig. 6*A*). To boost confirmation for a decrease in apoptosis as the mechanism of resistance, SW620 CV and KD and SW837 CV and KD cells were plated and underwent chemoradiation. Protein was collected after 24 h to assess for cleaved caspase 3. We found an increase in cleaved caspase 3 in the ST6GAL-1 KD cells compared with CV cells consistent with the findings of our caspase assay (N = 3) (Fig. 6*B*).

### Sialylation of TNFR1 is increased after chemoradiation

**Figure 7:**
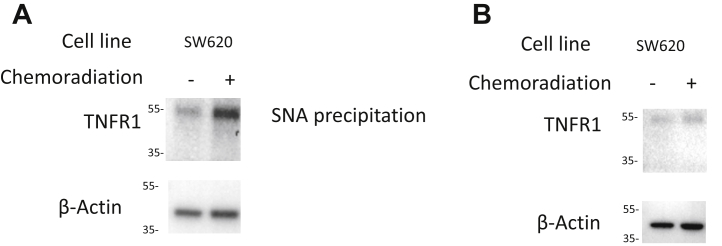
**ST6Gal-1 sialylation through TNFR1 mechanisms.***A*, lectin blot for TNFR1 in SW620 cells 24 h after treatment and immunoprecipitation with SNA revealed increased sialylated TNFR1 in the treated cells compared with untreated (2.3-fold increase, *p* = 0.02, N = 3). *B*, Western blot for total TNFR1 in SW620 cells 24 h after treatment revealed no change in TNFR1 between treatment groups (*p* = 0.22, N = 3). SNA, *Sambucus nigra*; TNFR1, tumor necrosis factor receptor 1.

In order to further investigate the mechanism of chemoradiation resistance because of ST6Gal-1, we interrogated a known target of sialylation after treatment. SW620 cell lines were treated with chemoradiation (3 μM of 5-FU and 5 Gy radiation) and collected 24 h later. We performed an SNA lectin precipitation to specifically collect sialylated proteins to investigate what proteins are altered by ST6GAL1 after treatment. We performed Western blotting of these sialylated proteins for TNFR1 because prior studies have shown that sialylation of TNFR1 causes the protein to be unable to signal apoptosis (7, 18). We found that chemoradiation-treated SW620 cells had a 2.3-fold increase in the sialylated form of TNFR1 after treatment (*p* = 0.02, N = 3; Fig. 7*A*). In addition, we collected all proteins 24 h after chemoradiation in SW620 cells, performed Western blotting for TNFR1, and found that there was not a significant difference between treatment groups when we assessed total TNFR1 (*p* = 0.22, N = 3; Fig. 7*B*).

## Discussion

Rectal cancer and its treatment result in major morbidity and mortality, but therapeutics have remained stagnant, likely because of poor understanding of the mechanism of resistance. Previous studies have shown that ST6GAL-1 is increased in various types of cancer, but the role of ST6GAL-1 in radiation resistance in rectal cancer has not been elucidated. We found that ST6GAL-1 and Sia on cell surface proteins are increased after chemoradiation in rectal cancer cell lines, rectal cancer–derived organoids, and PDX models using Western blot, flow cytometry, immunostaining, and immunoprecipitation. We also found that ST6GAL-1 appears to be subclonally expressed in individual rectal cancers *via* immunohistochemistry on our own patient samples. We then used rectal cancer cell lines to further elucidate the role of ST6GAL-1 in resistance to chemoradiation. KD of ST6GAL-1 confirmed its importance in blocking apoptosis and increasing survival after treatment. We also found that sialylated TNFR1 is increased after treatment, which prior studies have shown to decrease its ability to signal apoptosis. Consequently, we have shown that ST6GAL-1 may play a role in resistance to chemoradiotherapy in rectal cancer.

In the single publication assessing ST6GAL-1 in radiation resistance in cancer cells, Lee *et al.* (19) transduced the SW480 colon cancer cell line (which has low levels of ST6GAL-1 expression) with ST6GAL-1 and found that it became more resistant to radiation using a clonogenic assay, and that the cell surface protein β1-integrin in these cells had increased Sia after radiation treatment. They also found that ST6GAL-1 increased in the mouse spleen after whole animal radiation and that isolated proteins from the mouse spleen have increased Sia after radiation. Our study is the first to examine the role of ST6GAL-1 in resistance to chemoradiation in human rectal adenocarcinomas and PDX models and to assess the mechanism of resistance.

Our studies support the notion that ST6GAL-1 mediates resistance by decreasing apoptosis. There have been other studies that have investigated alterations in apoptosis as the mechanism of resistance mediated by ST6GAL-1 in other types of cancer. Holdbrooks *et al.* (7) found that ST6GAL-1 sialylates TNFR1 and protects tumor cells from TNF-induced apoptosis. In this study, they used KD or overexpression and found that high ST6GAL-1 expressers displayed resistance to apoptosis through decreased caspase 3 and 8 activity. They also found that TNFR1 internalization was inhibited by α2-6 sialylation. In addition, Swindall *et al.* (8) identified that the death receptor, Fas (CD95), is another ST6GAL-1 substrate and that sialylation of this receptor confers protection against Fas-mediated apoptosis. Interestingly, there was no effect on DR4 or DR5, indicating selective effect of ST6GAL-1 on Fas receptor. They also used ST6GAL-1 KD and forced overexpression with colon carcinoma cancer cell models and determined that sialylation of Fas prevented apoptosis as evidenced by decreased activation of caspase 3 and 8. Similarly, Alexander *et al.* (18) showed increased ST6GAL-1 expression in gastric premalignancy and adenocarcinoma, that ST6GAL-1 overexpression enhances resistance to TNF-induced epithelial cell apoptosis, and that ST6GAL-1 overexpression dysregulates apoptosis in gastric cancer. These prior studies did not assess rectal cancer cell lines or human tumors and did not assess the effects of chemoradiation; however, it appears likely that ST6GAL-1 is inhibiting apoptosis caused by chemoradiation *via* TNFR1. We found that sialylated TNFR1 is increased after treatment, and prior studies have shown that this sialylation causes a change in the protein such that it is not internalized and thus does not signal apoptosis.

Little is known regarding mechanisms of resistance in rectal cancer. It is well recognized that rectal cancer is genetically heterogeneous with potentially multiple mechanisms of therapeutic resistance across tumors (20, 21). Previous studies have identified particular markers that may be utilized to predict response, but these have yet to be validated in larger studies (22, 23). Notable pathways appear to be DNA repair, apoptosis, and the immune response (24). We have identified a potential mechanism of resistance to radiation-induced apoptosis *via* ST6GAL-1.

Our study has some potential limitations. We did not assess ST6GAL-1 mRNA or protein in matched pre-*versus*-post treatment human rectal cancer specimens because of lack of availability of these specimens. We assessed mRNA for ST6GAL-1 in untreated patient rectal cancer samples and did not find it to be elevated compared with normal rectum, but there are some potential issues with interpreting these data. First, ST6Gal-1 is found in multiple immune cells so bulk RNA-Seq of tumor tissue will reflect ST6Gal-1 in immune cells as well as the tumor cells making these data more difficult to interpret (15, 25). Second, prior studies have suggested that ST6Gal1 mRNA levels do not always correspond directly with ST6Gal1 protein expression or enzymatic activity (26, 27). Notably, ST6Gal1 expression is heavily regulated at the post-transcriptional level through processes including translational regulation (28) and suppression of ST6Gal1 expression through miRNAs (6). In addition, while we have shown that ST6GAL-1 appears to likely alter radiation resistance *via* sialylation of proteins that mediate apoptosis in rectal cancer cell lines, but there are other possible mechanisms of resistance *via* sialylation or otherwise. The number of organoid samples and PDX samples we investigated were low because of availability. ST6GAL-1 is likely not responsible for resistance in every rectal cancer, but our data show that it warrants further investigation. Finally, we did not investigate whether ST6GAL-1 in pretreatment human rectal cancer samples correlates with increased ST6GAL-1 after chemoradiation or with increased ST6GAL-1-mediated resistance to chemoradiation.

### Conclusions

We have shown that ST6GAL-1 mediates resistance to chemoradiotherapy in rectal cancer cell lines and increases after treatment in some PDX and organoid rectal cancer models and that it is present in some untreated human rectal adenocarcinomas. Our data also indicate that the therapeutic resistance in cell lines is likely *via* inhibition of apoptosis. More research is needed to further elucidate the importance of ST6GAL-1-mediated resistance and its mechanism in human rectal cancer.

## Experimental procedures

### Human samples

For immunostaining, the histology of paraffin-embedded rectal adenocarcinoma and adjacent normal tissue was assessed by a gastrointestinal pathologist (S. A. D.) and provided by the institutional Tissue Biorepository as part of our institutional review board–approved protocol. For RNA samples, tissue biopsies were obtained from rectal adenocarcinomas and adjacent normal rectal tissue in consented patients according to our institutional review board protocol prior to undergoing treatment and frozen in optimal cutting temperaure compound. Patient-derived xenograft models were created when adequate tissue was available.

### Xenograft studies

Patient-derived xenografts were created from biopsies from consented patients with rectal cancer prior to treatment. All animal protocols were performed in accordance with our institutional-approved protocol. Fresh tumor samples were rinsed with PBS and gentamycin (Thermo Fisher Scientific); necrotic tissue was removed, and a 2 mm^3^-sized tumor piece was implanted into Central Institute for Experimental Animals NOG mice (Taconic). Once tumors were of appropriate size (10 × 20 mm^3^), mice were sacrificed, and tumors were removed. Tumor pieces of 2 mm^3^ were implanted in the flank of Nude mice (Jackson Laboratories). Once tumors reached 150 mm^3^, mice were split into the following treatment groups: vehicle or capecitabine (100 mg/kg) and 2 Gy (Gy) radiation 5 days per week for 2 weeks. Capecitabine (LC Laboratory) was given *via* oral gavage. Mice were placed in a custom jig for shielding, and radiation was delivered to tumors *via* X-RAD 320 (Precision X-Ray, Inc) daily. Mice were weighed, and tumors were measured with calipers at least twice weekly. Tumor volumes were calculated based on caliper measurements as previously described (29). Tumors were harvested 1 week after completion of the 2-week treatment.

### Organoids

Tumors were excised from mice and dissociated using 2 mg/ml collagenase I (Thermo Fisher Scientific). Resulting dissociated tissue and cells were filtered using a 70 μm nylon cell strainer and centrifuged at 250*g* for 5 min at room temperature. Viability and concentration were assessed using a Countess II Cell Counter (Thermo Fisher Scientific). Cells were plated at 50,000 live cells in 15 μl Matrigel Matrix (Thermo Fisher Scientific). Once Matrigel was solidified, cells were cultured with organoid media consisting of 50% advanced Dulbecco's modified Eagle's medium (DMEM; Thermo Fisher Scientific), 50% L-WRN conditioned media (American Type Culture Collection [ATCC] CRL-3276; both supplemented with 20% fetal bovine serum (FBS) (Sigma), 2 mM GLUTamax, 100 units/ml penicillin and 0.1 mg/ml streptomycin, 10 μM SB 431542, 10 μM Y-27632, and 50 μg/ml gentamicin (all Thermo Fisher Scientific)). Media were changed every other day.

### Cell culture and treatment with chemoradiation

The SW837 (CCL-235) cell line was obtained from ATCC. The SW620 (CCL-227) cell line was donated by the Leopold laboratory at the University of Michigan. The SW837 cell line was grown in advanced DMEM with 5% FBS with 100 units/ml penicillin and 0.1 mg/ml streptomycin and 0.25 μg/ml fungizone and 2 mM GLUTamax (Thermo Fisher Scientific), and SW620 cells were grown in DMEM with 10% FBS with 100 units/ml penicillin and 0.1 mg/ml streptomycin and 0.25 μg/ml fungizone (Thermo Fisher Scientific). Cell lines were verified by short tandem repeat testing on samples of cultured cells sent to ATCC at regular intervals. Cell were used at low passage numbers only. For chemoradiation treatment of cell lines, cells were treated with 5-FU (Selleckchem) (3 μM) 30 min prior to radiation (5 Gy) (IC-320; Kimtron Medical), and media were changed the following day.

### ST6GAL-1 and Sia immunostaining

Human rectal adenocarcinoma tissue slides were deparaffinized and rehydrated by washing in xylene and ethanol, and the slides were then placed in boiling citric acid–based antigen unmasking solution (Vector Labs). Slides were washed and then covered in BLOXALL solution (Vector Labs) for 5 min and 0.5% Triton-X in PBS for 25 min. The slides were then blocked with 2.5% horse serum and incubated with primary antibody against ST6GAL-1 1:100 overnight (R&D Systems). ImmPRESS horse antigoat immunoglobulin G (IgG) (Vector Labs) was used as the secondary antibody, with NovaRED peroxidase substrate (Vector Labs) used for the primary stain for 2 min. Slides were counterstained with hematoxylin for 30 s and paraffinized. We assessed immunostaining in four human rectal adenocarcinomas.

For immunofluorescence studies, SW620 and SW837 rectal cancer cells and organoids were plated onto coverslips, treated with chemoradiation, washed, and then fixed for 15 min with 4% paraformaldehyde. Slides were washed and permeabilized using PBS with Tween-20 + 0.5% Triton X-100, cells for 10 min, and organoids for 30 min. Slides were blocked with PBS with Tween-20 + 3% bovine serum albumin. Primary ST6GAL-1 (1:150) antibody (R&D Systems) was applied followed by donkey antigoat IgG secondary (1:500) antibody (Invitrogen). SNL conjugated to FITC was used for staining of Sia (Vector Labs) and was added for 1 h, and then 4′,6-diamidino-2-phenylindole nuclear stain (Invitrogen) was placed in blocking buffer, and slides were incubated. Slides were mounted with VectaMount (Vector Labs) and then imaged.

### qPCR

RNA was extracted from previously frozen rectal adenocarcinoma and matching normal rectal tissue samples or rectal cancer organoids using the RNeasy kit (Qiagen), and equal amounts of RNA were used to generate complementary DNA with the High-Capacity cDNA Reverse Transcription Kit (Fisher). qPCR was performed using a QuantStudio3 (Thermo Fisher Scientific) instrument. TaqMan gene expression assay primers (Thermo Fisher) and TaqMan Fast Advanced Master Mix (Thermo Fisher) were used to generate reactions.

### Lentivirus transduction for ST6GAL-1 KD

SW620 and SW837 cells were plated with 50,000 cells per well in a 24-well plate and allowed to incubate overnight. Cells were washed with PBS and then lentivirus with shRNA for ST6GAL-1 to cause ST6GAL-1 KD (KD, sequence CCGGCGTGTGCTACTACTACCAGAACTCGA GTTCTGGTAGTAGTAGCACACGTTTTTG) or CV (nonmammalian-targeted shRNA, both donated by the Bellis laboratory) was added to each well to obtain a multiplicity of infection of five in serum-free antibiotic-free media. Cells were incubated overnight, media were removed, cells were washed with PBS, and cultured in complete media for 48 h. Cells were cultured in puromycin (1 μg/ml) selection media for 7 days. KD was confirmed by Western blotting for ST6GAL-1 and flow cytometry for Sia using the SNA lectin as described.

### Western blotting

SW620 and SW837 cells were lysed in radioimmunoprecipitation assay buffer and Halt protease and phosphatase inhibitor cocktail (Fisher Scientific) 1 or 5 days after treatment. PDX model tumors were harvested and dissociated 1 week after completion of treatment. Samples then underwent electrophoresis and protein transfer to polyvinylidene fluoride membranes. Membranes were blocked in 5% nonfat dry milk in 1× Tris-buffered saline and 0.1% Tween-20. Blots were probed with 1:1000 antibodies against ST6GAL-1 (R&D Systems), (1:200) cleaved caspase 3 (Cell Signaling Technology), and (1:300) TNFR1 (Cell Signaling Technology). Protein loading was verified using 1:10,000 β-actin (Abcam). Membranes were incubated with horse antigoat IgG secondary antibody for ST6GAL-1 (R&D Systems) and biotinylated rabbit secondary for cleaved caspase 3 (Vector Labs) and rabbit monoclonal antibody for TNFR1 (Santa Cruz) and imaged using enhanced chemiluminescence.

### Apoptosis assay

Cells were plated at equal densities into a 96-well plate. Cells were treated 24 h after plating with 5-FU (3 μM) and radiation (5 Gy), and 24 h after treatment, Caspase-Glo 3/7 Assay reagent (Promega) was added in equal amounts to the media in each well. Plates were incubated for 1 h at room temperature, and luminescence was quantified with Infinite M200 Pro instrument (Tecan).

### Colony formation assay

SW620 cells were plated at 60% confluence and then treated 24 h later with 5-FU (3 μM) and radiation (5 Gy). Post-treatment, cells were trypsinized and then diluted to plate 500 cells per well in 6-well plates. Following plating, cells were grown in appropriate serum until colonies were visible. Cells were washed with PBS and then stained with 0.1% crystal violet for 10 min. Colonies containing over 50 cells were counted.

### Flow cytometry

SW620 and SW837 cells were washed in a fluorescence-activated cell sorting (FACS) buffer (1× PBS + 2% FBS), filtered, and resuspended in FACS buffer. Cells were counted and labeled with FITC-labeled SNA at 20 μg/ml of SNA lectin per 10^6^ cells. Cells were incubated in the dark for 45 min, washed, and resuspended with FACS buffer. Propidium iodide was added (1 μg/200 μl). Flow cytometry was performed at the University of Alabama FACS facility.

### SNA precipitation

SW837 cells were plated until 80% confluence and then lysed in radioimmunoprecipitation assay buffer and protease/phosphatase inhibitors 24 h after treatment, and protein was collected. About 50 μl of SNA-agarose beads (Vector Labs) were washed and then incubated overnight with 500 μg of the collected protein. Sample was pelleted, washed, and resuspended in wash buffer. Protein was removed from the beads using glycoprotein eluting buffer (Vector Labs) or heating in 1× sample buffer with beta-mercaptoethanol. Samples then underwent electrophoresis and protein transfer to polyvinylidene fluoride membranes, membranes were blocked in 5% nonfat dry milk in 1× Tris-buffered saline and 0.1% Tween-20. Membranes were probed for SNA (1:1000) with SNA, elderberry lectin, biotinylated (Vector), and incubated with secondary (1:300) with streptavidin peroxidase. Protein loading was verified using 1:10,000 β-tubulin antibody (Abcam).

### Statistical analysis

Western blots were quantified and normalized to control protein using ImageJ (NIH, version 1.53a) and analyzed using a Student’s *t* test for statistical comparison. qPCR was analyzed using the comparative CT method (18). For the apoptosis assay, measurements of relative light units at 5 Gy for each cell type were converted to fold change values by dividing each 5 Gy measurement by the corresponding 0 Gy mean value of the same cell type (KD or CV) within each experiment. Subsequently, the log fold change was fit using a linear mixed model with cell type treated as a fixed effect and experiment as a random intercept to account for correlation in technical replicates. The model thus assessed for a differential change in apoptosis with radiation because of knock down of ST6Gal1 as compared with control. For analysis of flow cytometry data, measured values following treatment consisted of number of cells positive for SNA as well as mean fluorescent intensity. The number of SNA-positive cells and total cells was fit separately for SW620 and SW837 cell lines using a logistic regression mixed model with radiation treatment as a fixed effect and experiment as a random intercept. The model thus assessed the effect of radiation on the odds of SNA-positive cells for both cell lines. In addition, mean fluorescent intensity data collected for each cell line in FACS experiments were analyzed by paired *t* test to evaluate the hypothesis of no change in log fold change in intensity for radiation treatment from 0 Gy and 5 Gy. Finally, colony formation was analyzed by converting colony counts to fold change values by dividing each 5G measurement corresponding the 0G mean value of the same cell type (KD or CV) within each experiment (n = 3 experiments per cell type). Subsequently, the log fold change was fit using a linear mixed model with cell type (KD *versus* CV) as the fixed effect and a random intercept for experiment to account for correlation among technical replicates. Experiments in cell lines were replicated using different passages at least three separate times. Data cleaning and analysis utilized R software, version 4.0.2 (R Core Team, 2020). All mixed models utilized restricted maximum likelihood estimation and the R package lme4 (version 1.1-23). Statistical tests were two-sided with significance at *p* < 0.05.

## Data availability

All data are contained within the article.

## Conflict of interest

M. S. reports that financial support was provided by the 10.13039/100000051National Human Genome Research Institute. K. M. H. reports that financial support was provided by the 10.13039/100000002National Institutes of Health. All other authors declare that they have no conflicts of interest with the contents of this article.
